# Short-term outcome of isolated lateral malleolar fracture treatment is independent of hospital trauma volume or teaching status: a nationwide retrospective cohort study

**DOI:** 10.1007/s00068-021-01771-4

**Published:** 2021-08-16

**Authors:** Malte Vehling, Claudio Canal, Franziska Ziegenhain, Hans-Christoph Pape, Valentin Neuhaus

**Affiliations:** Division of Trauma Surgery, Department of Traumatology, University Hospital Zurich, University of Zurich, Raemistrasse 100, CH-8091 Zurich, Switzerland

**Keywords:** High-volume, Low-volume, Malleolar fracture, Orthopaedic trauma, Outcome

## Abstract

**Introduction:**

In light of current discussions about centralisation and teaching in medicine, we wanted to investigate the differences in in-hospital outcomes after surgical treatment of isolated ankle fractures, taking into account high-volume centres (HVCs) and low-volume centres (LVCs) and teaching procedures.

**Methods:**

A retrospective analysis of malleolar fractures recorded in a National Quality Assurance Database (AQC) from the period 01-01-1998 to 31-12-2018 was carried out. Inclusion criteria were isolated, and operatively treated lateral malleolar fractures (ICD-10 Code S82.6 and corresponding procedure codes). Variables were sought in bivariate and multivariate analyses. A total of 6760 cases were included. By dividing the total cases arbitrarily in half, 12 HVCs (*n *= 3327, 49%) and 56 LVCs (*n *= 3433, 51%) were identified.

**Results:**

Patients in HVCs were younger (48 vs. 50 years old), had more comorbidities (26% vs. 19%) and had more open fractures (0.48% vs. 0.15%). Open reduction and internal fixation was the most common operative treatment at HVCs and LVCs (95% vs. 98%). A more frequent use of external fixation (2.5% vs. 0.55%) was reported at HVCs. There was no difference in mortality between treatment at HVCs and LVCs. A longer hospitalisation of 7.2 ± 5 days at HVCs vs. 6.3 ± 4.8 days at LVCs was observed. In addition, a higher rate of complications of 3.2% was found at HVCs compared to 1.9% at LVCs. The frequency of teaching operations was significantly higher at HVCs (30% vs. 26%). Teaching status had no influence on mortality or complications but was associated with a prolonged length of stay and operating time.

**Conclusion:**

We found significant differences between HVCs and LVCs in terms of in-hospital outcomes for ankle fractures. These differences could be explained due to a more severely ill patient population and more complex (also open) fracture patterns with resulting use of external fixation and longer duration of surgery. However, structural and organisational differences, such as an extended preoperative stays at HVCs and a higher teaching rate, were also apparent. No difference in mortality could be detected.

## Introduction

Ankle fractures are common with an incidence of 122 to 187 per 100,000 population and account for the majority of foot and ankle injuries [1, 2]. They account for 10–12% of all fractures [3, 4]. Because of this high incidence, ankle fractures are not only a relevant medical topic but also one that requires attention from an economic point of view. Therefore, ankle fractures are ideal for research purposes.

Centralisation is already state of the art in highly specialised medical disciplines and has become entrenched by improving care and enhancing treatment outcomes [5]. Currently, there is both political and social debate about the need for centralisation in other areas of medical care [6–8]. This debate is being driven forward not only by medical aspects but also by economic and political considerations [9]. However, the implementation of centralisation and its extension to less highly specialised treatments has not yet been conclusively resolved in the medical community [10, 11]. In this context, there is a lack of valid data to compare the outcome of malleolar fractures between high-volume centres (HVCs) and low-volume centres (LVCs). Apart from the aspect of centralisation, teaching has an important influence on outcome [12]. Treating ankle fractures is a standard teaching procedure for surgical residents due to the high incidence and standardised treatment.

The aim of this study was to compare in-hospital outcomes in terms of mortality, complication rate and length of stay (LOS) for a common injury with surgical treatment between HVCs and LVCs. The possible influence of teaching with the in-hospital outcome was also evaluated in this context.

## Methods

### Study design and setting

The national database of the Swiss Working Group for Quality Assurance in Surgery (Arbeitsgemeinschaft für Qualitätssicherung in der Chirurgie, AQC) was used for data collection. The AQC is an association for prospective data collection and scientific data analyses regarding surgical interventions and diseases. Membership and data entry is voluntary for surgical departments, however the Swiss Surgical Association gives a recommendation to use it. Since the AQC was established in 1995, more than 80 Swiss surgical departments have entered over 1.5 million cases into the database, this accounts for 85% of all Swiss surgical departments [13, 14]. The AQC database provides useful, prospective in-hospital data for scientific studies [15–17]. Using this database, a retrospective analysis of patients who suffered isolated lateral malleolar fractures between 1998 and 2018 was performed.

### Participants/Study subjects

We included only patients with an isolated malleolar fracture who were treated operatively between 1998 and 2018. To identify these patients, we used the corresponding World Health Organization (WHO) International Classification of Disease Code (ICD-10) [18] S82.6 and the corresponding Schweizerische Operationsklassifikation procedure code (CHOP Code) [19] 79.36 with its subcategories. Additionally, a complete AQC form must have been available. These criteria were met by 6760 data sets after excluding 1813 patients due to incomplete AQC forms. In this study we used only anonymised data, therefore no additional ethical approval was required according to the local cantonal ethical review board (KEK, Zurich, Switzerland) [20]. Patients must however be informed and give general consent to enter their data prospectively into the AQC database.

### Variables, outcome measures and data sources

During the study period, 69 surgical units provided data for patients with lateral malleolar fractures via the AQC system. The AQC data contain information on patient age and gender, specifications about the type of admission (registered/planned or emergency), insurance status (private or statutory), length of hospital stay, time in the intensive care unit (ICU), concomitant injuries, American Society of Anesthesiologists (ASA) score (physical status classification system), comorbidities, need for reintubation and information about the discharge status (death, home, nursing home, senior residence, rehabilitation clinic and others). Moreover, the following procedural characteristics were available: fracture site (classified according to ICD-10 [18]); education level of the surgeon (divided into three groups—senior attending, junior attending (fellow) and residents); teaching status of the operation; procedure code (coded according to the CHOP code [19]) and duration of the surgery; other interventions; presence and severity of any perioperative complications (no complications, complications with conservative therapy, complications with surgical therapy, complications with long-term damage with conservative therapy and mortality); and the use of thromboembolism prophylaxis and antibiotics [12].

The hospitals providing data for this study were divided into two groups solely based on the number of ankle fracture cases treated surgically during the study period. By dividing the total cases arbitrarily in half, 12 HVCs (*n *= 3327, 49%; HVC) and 56 LVCs (*n *= 3433, 51%; LVC) were identified. Mortality, complication rates, and LOS were evaluated as outcome parameters. Independent variables were, among others, HVCs vs. LVCs and teaching status of the procedures. The definition of HVC/LVC is arbitrary—to minimize the grey zone for quantitative differences, we added a subanalysis: the highest 10% (2 centres with 704 patients) and lowest 10% (29 centres with 667 patients) were examined separately.

### Statistical analysis

We extracted our data online from the AQC database using the evaluation tool AdjumedAnalyze (Adjumed Services AG, Zurich, Switzerland) and used the Statistical Package for Social Sciences (SPSS, version 24; IBM Corp., Armonk, NY, USA) for our statistical analysis. Chi-square, Mann–Whitney *U* and Fisher tests were used where applicable for bivariate analysis. We found no normal distribution in our data based on a Kolmogorov–Smirnov test. Significant (*p *< 0.05) or nearly significant factors (*p *< 0.1) in bivariate analysis were chosen as potential risk factors for complications. These risk factors were evaluated as confounders in a stepwise backward likelihood logistic regression analysis. We performed only bivariate analysis with predictors for mortality due to the low number of deaths (*n *= 15). A *p* value of < 0.05 was considered statistically significant.

## Results

### Demographics

On average, 277 patients (209–382) were treated at HVCs, while 61 patients (1–176) were treated at LVCs. Patients at HVCs were younger (48 vs. 50 years old), predominantly male (52% vs. 48%), had a higher ASA category on average (ASA I 54% vs. 57%, ASA II 41% vs. 37%) and had more comorbidities (26% at HVCs and 19% at LVCs). However, patients categorised as ASA III–V were more often treated at LVCs (5.2%, 0.58%, 0.55% at LVCs vs. 4.1%, 0.06%, 0% at HVCs). The rate of emergency and planned admissions were similar between HVCs and LVCs. The rate of privately insured patients was higher at HVCs (29% vs. 24%). A higher number of open fractures (oFx) were treated at HVCs (*n *= 16, 0.48% at HVCs vs. *n *= 5, 0.15% at LVCs) (Table 1).

**Table 1:** High-volume vs. low-volume; patient characteristics

Parameter	Total (*n *= 6760)		High-volume (*n *= 3327)		Low-volume (*n *= 3433)		*p* value
	*n*	%	*n*	%	*n*	%	
Age (years)							
Mean ± SD	49 ± 18		48 ± 18		50 ± 18		< 0.001
Gender							
Male	3392	50	1744	52	1648	48	< 0.001
Female	3368	50	1583	48	1785	52	
ASA							
I	3767	56	1813	54	1954	57	< 0.001
II	2640	39	1377	41	1263	37	
III	312	4.6	135	4.1	177	5.2	
IV	22	0.33	2	0.060	20	0.58	
V	19	0.28	0	0	19	0.55	
Comorbidity							
Yes	1511	22	865	26	646	19	< 0.001
Admission type							
Emergency	5676	84	2776	83	2900	84	n.s
Registered, planned	1084	16	551	17	533	16	
Insurance							
Statutory	4949	73	2349	71	2600	76	< 0.001
Private	1811	27	978	29	833	24	
Open fracture							
Yes	21	0.31	16	0.48	5	0.15	0.013
Length of stay (days)							
Mean ± SD	6.7 ± 4.9		7.2 ± 5.0		6.3 ± 4.8		< 0.001
Length of stay preoperative (days)							
Mean ± SD	1.8 ± 2.3		2.2 ± 2.4		1.5 ± 2.2		< 0.001
Length of stay postoperative (days)							
Mean ± SD	4.9 ± 4.0		5.1 ± 4.1		4.8 ± 4.0		< 0.001
Duration ICU (hours)							
Mean ± SD	0.15 ± 3.6		0.078 ± 1.6		0.22 ± 4.7		n.s
Complications							
Yes	171	2.5	105	3.2	66	1.9	0.001
Discharge							
Deceased	15	0.22	5	0.15	10	0.29	n.s
At home	6248	92	3091	93	3157	92	
Nursing home	51	0.75	18	0.54	33	0.96	
Old people’s home	49	0.72	19	0.57	30	0.87	
Rehabilitation clinic	185	2.7	82	2.5	103	3.0	
Other	212	3.1	112	3.4	100	2.9	

*ASA* American Society of Anesthesiologists Physical Status Classification, *ICU* Intensive Care Unit, *SD* Standard Deviation

### Treatment

The most common surgical treatment was open reduction and internal fixation (ORIF) (96%), followed by external fixation (1.5%). ORIF was less frequently used at HVCs compared to LVCs (95% vs. 98%), while external fixation was more frequently used at HVCs (2.5% vs. 0.55%). In the subgroup of oFx treatment, external fixation was more common (33% in the oFx subgroup vs. 1.4% in the closed fracture (cFx) subgroup; *p *< 0.001). Duration of surgery was slightly longer at HVCs (65 ± 32 min at HVCs vs. 61 ± 28 min at LVCs). Antibiotics were administered in most cases (89%). The usage of prophylactic antibiotics was more common at HVCs (94% at HVCs vs. 83% at LVCs). All patients with an oFx received antibiotics (Table 2).

**Table 2:** High-volume vs. Low-volume; procedure characteristics

Parameter	Total (*n *= 6760)		High-volume (*n *= 3327)		Low-volume (*n *= 3433)		*p* value
	*n*	%	*n*	%	*n*	%	
Surgeon class							
Senior attending	2603	39	983	30	1620	47	< 0.001
Junior attending	2413	36	1345	40	1068	31	
Resident	1744	26	999	30	745	22	
Type of surgery							
ORIF	6523	96	3146	95	3377	98	< 0.001
External fixator	101	1.5	82	2.5	19	0.55	
Suture capsule or ligament	51	0.75	43	1.3	8	0.23	
Hardware removal	32	0.47	21	0.63	11	0.32	
CR	34	0.50	24	0.72	10	0.29	
CRIF	17	0.25	11	0.33	6	0.17	
Other reconstruction of the ankle joint	2	0.030	0	0	2	0.058	
Duration surgery (minutes)							
Mean ± SD	63 ± 30		65 ± 32		61 ± 28		< 0.001
Teaching							
Yes	1897	28	996	30	901	26	< 0.001
Antibiotics							
No antibiotics	761	11	177	5.3	584	17	< 0.001
Prophylactic antibiotics (before start)	5841	86	3054	92	2787	81	
Prophylactic antibiotics (after start)	106	1.6	60	1.8	46	1.3	

*CR* Closed Reduction, *IF* Internal Fixation, *OR* Open Reduction, *SD* Standard Deviation

### Outcome

Overall, the complication rate was 2.5% (3.2% at HVCs vs. 1.9% at LVCs) (Table 1). We found an almost quadruple rate of complications in oFx compared to cFx (9.5% vs. 2.5%; *p *= 0.041). The most common complications were disturbance of wound healing, secondary dislocation, malpositioning of the implants, lesion to the nerve and hematoma. Predictors for complications were age, ASA III–IV, junior attending vs. senior attending, duration of surgery and hospital volume (Table 3). Preoperative LOS had no influence. On average, the LOS was 6.7 ± 4.9 days (7.2 ± 5 days at HVCs and 6.3 ± 4.8 days at LVCs). A significantly longer pre- and postoperative stay was observed at HVCs. ICU stays were very rare, lasting 0.15 ± 3.6 h when they did occur (even shorter duration at HVCs), and had no statistical significance (Table 1). A significantly longer hospital stay was noticed for the oFx subgroup (15 ± 6.9 for the oFx subgroup vs. 6.7 ± 4.9 for the cFx subgroup, *p *< 0.001). Most patients were discharged home (92%), followed by discharge to a rehabilitation clinic (2.7%), nursing home (0.75%) or senior residence (0.72%); this was similar for HVCs and LVCs. Fifteen patients died in hospital (0.22%; 0.15% at HVCs and 0.29% at LVCs) (Table 1).

**Table 3:** Predictors complications

	Sig	OR	95% C.I. for OR	
			Lower	Upper
Age	** < 0.001**	1.021	1.011	1.031
ASA II (vs. I)	0.963	0.992	0.699	1.408
ASA III (vs. I)	**0.021**	1.953	1.108	3.444
ASA IV (vs. I)	**0.039**	5.144	1.082	24.447
Junior attending (vs. senior attending)	**0.019**	1.544	1.073	2.220
Resident (vs. senior attending)	0.850	0.959	0.620	1.483
Duration surgery	** < 0.001**	1.010	1.006	1.014
Prophylactic antibiotics (before start) (vs. no antibiotics)	0.856	1.054	0.597	1.861
Prophylactic antibiotics (after start) (vs. no antibiotics)	0.919	1.069	0.297	3.855
Antibiotic therapy (vs. no antibiotics)	**0.003**	4.524	1.677	12.203
High-volume hospital (vs. low-volume)	**0.006**	1.579	1.141	2.184

Bold values indicate Statistically significant

*ASA* American Society of Anesthesiologists Physical Status Classification, *C.I.* confidence interval, *OR* Odds Ratio

### Teaching

A total of 28% of the operations were performed as teaching procedures. The rate was significantly higher at HVCs (HVCs 30% vs. LVCs 26%) (Table 2). Teaching was not associated with a higher mortality rate (0.16% in teaching yes vs. 0.047% in teaching no) or a higher complication rate (2.8% in teaching yes vs. 2.9% in teaching no). However, a significantly longer operation time (68 ± 29 in teaching yes vs. 59 ± 30 in teaching no) and a prolonged LOS (6.9 ± 5 in teaching yes vs. 6.2 ± 4.7 in teaching no) were observed (Table 4).

**Table 4:** Teaching YES vs. NO

Parameter	Total (*n *= 6760)		Teaching NO (*n *= 2112)		Teaching YES (*n *= 1897)		*p* value
	*n*	%	*n*	%	*n*	%	
Age (years)							
Mean ± SD	49 ± 18		50 ± 18		48 ± 18		< 0.001
Gender							
Male	3392	50	1034	49	1013	53	0.005
Female	3368	50	1078	51	884	47	
ASA							
I	3767	56	1121	53	1078	57	0.008
II	2640	39	910	43	722	38	
III	312	4.6	79	3.7	95	5.0	
IV	22	0.33	2	0.095	1	0.053	
V	19	0.28	0	0	1	0.053	
Comorbidity							
Yes	1511	22	503	24	408	22	n.s
Admission type							
Emergency	5676	84	1724	82	1579	83	n.s
Registered, planned	1084	16	388	18	318	17	
Insurance							
Statutory	4949	73	1340	63	1659	88	< 0.001
Private	1811	27	772	37	238	13	
Length of stay (days)							
Mean ± SD	6.7 ± 4.9		6.2 ± 4.7		6.9 ± 5.0		< 0.001
Length of stay preoperative (days)							
Mean ± SD	1.8 ± 2.3		1.7 ± 2.3		2.0 ± 2.3		< 0.001
Length of stay postoperative (days)							
Mean ± SD	4.9 ± 4.0		4.6 ± 3.8		4.9 ± 4.0		< 0.001
Duration ICU (hours)							
Mean ± SD	0.15 ± 3.6		0.055 ± 1.1		0.067 ± 1.1		n.s
Complications							
Yes	171	2.5	62	2.9	54	2.8	n.s
Discharge							
Deceased	15	0.22	1	0.047	3	0.16	n.s
Duration surgery (minutes)							
Mean ± SD	63 ± 30		59 ± 30		68 ± 29		< 0.001

*ASA* American Society of Anesthesiologists Physical Status Classification, *ICU* Intensive Care Unit, *SD* Standard Deviation

### Subgroup analysis

Patients in the 10% highest volume centres were slightly older (50 vs. 48 years), sicker (33% vs. 26% comorbidities), more often admitted as planned surgery (24% vs. 17%), more often operated by junior attendings (45% vs 40%) as a teaching procedure (43% vs. 30%) within longer time of surgery (76 vs 65 min) than in the other HVC. ORIF was the primary treatment in 90% (vs. 95%). The length of stay and outcome were similar.

Patients in the 10% lowest volume centres were quite similar with the other LVC cohort.

The subgroup analysis of the top-10% and low-10% centres confirmed the results of the HVC vs. LVC evaluation—except for age (Tables 5 and 6).

**Table 5:** High-volume vs. low-volume; patient characteristics

Parameter	Total (*n *= 6760)		10% High-volume (*n *= 704)		10% Low-volume (*n *= 667)		*p* value
	*n*	%	*n*	%	*n*	%	
Age (years)							
Mean ± SD	49 ± 18		50 ± 18		49 ± 18		0.04
Gender							
Male	3392	50	392	56	317	48	0.003
Female	3368	50	312	44	350	53	
ASA							
I	3767	56	440	63	414	62	n.s
II	2640	39	235	33	222	33	
III	312	4.6	29	4.1	30	4.5	
IV	22	0.33	0	0	1	0.15	
V	19	0.28	0	0	0	0	
Comorbidity							
Yes	1511	22	233	33	132	20	0.002
Admission type							
Emergency	5676	84	538	76	568	85	< 0.001
Registered, planned	1084	16	166	24	99	15	
Insurance							
Statutory	4949	73	516	73	536	80	0.002
Private	1811	27	188	27	131	20	
Open fracture							
Yes	21	0.31	7	0.13	2	0.30	0.01
Length of stay (days)							
Mean ± SD	6.7 ± 4.9		7.3 ± 4.2		6.8 ± 4.9		0.048
Length of stay preoperative (days)							
Mean ± SD	1.8 ± 2.3		2.4 ± 2.4		1.4 ± 2.2		< 0.001
Length of stay postoperative (days)							
Mean ± SD	4.9 ± 4.0		4.9 ± 3.3		5.4 ± 4.3		0.027
Duration ICU (hours)							
Mean ± SD	0.15 ± 3.6		0 ± 0		0.0090 ± 0.094		0.012
Complications							
Yes	171	2.5	24	3.4	10	1.5	0.023
Discharge							
Deceased	15	0.22	0	0	1	0.15	< 0.001
At home	6248	92	603	86	618	93	
Nursing home	51	0.75	7	0.99	2	0.30	
Old people’s home	49	0.72	1	0.14	5	0.75	
Rehabilitation clinic	185	2.7	21	3.0	14	2.1	
Other	212	3.1	72	10	27	4.0	

*ASA* American Society of Anesthesiologists Physical Status Classification, *ICU* Intensive Care Unit, *SD* Standard Deviation

**Table 6:** High-volume vs. low-volume; procedure characteristics

Parameter	Total (*n *= 6760)		10% high-volume (*n *= 704)		10% low-volume (*n *= 667)		*p* value
	*n*	%	*n*	%	*n*	%	
Surgeon class							
Senior attending	2603	39	164	23	308	46	< 0.001
Junior attending	2413	36	315	45	204	31	
Resident	1744	26	225	32	155	23	
Type of surgery							
ORIF	6523	96	635	90	656	98	< 0.001
External fixator	101	1.5	42	6.0	1	0.15	
Suture capsule or ligament	51	0.75	5	0.71	6	0.90	
Hardware removal	32	0.47	9	1.3	1	0.15	
CR	34	0.50	6	0.85	1	0.15	
CRIF	17	0.25	7	0.99	2	0.30	
Other reconstruction of the ankle joint	2	0.030	0	0	0	0	
Duration surgery (minutes)							
Mean ± SD	63 ± 30		76 ± 37		66 ± 31		< 0.001
Teaching							
Yes	1897	28	305	43	167	25	< 0.001
Antibiotics							
No antibiotics	761	11	14	2.0	119	18	< 0.001
Prophylactic antibiotics (before start)	5841	86	681	97	533	80	
Prophylactic antibiotics (after start)	106	1.6	1	0	11	1.6	

*CR* Closed Reduction, *IF* Internal Fixation, *OR* Open Reduction, *SD* Standard Deviation

## Discussion

With the ongoing political and socio-economic debate about medical infrastructure and the necessity of smaller clinics vs. the importance of the centralisation of medical specialties, we took a closer look at the short-term in-hospital outcomes of isolated malleolar fractures in Swiss hospitals [8]. Ankle fractures are among the most common bone fractures in Switzerland [21, 22]. According to Schweizerische Unfallversicherungsanstalt (SUVA) statistics, the subcategory of lower leg, ankle and foot fractures accounts for roughly a third of the total cost of fracture management [21].

For this study, we collected data from the AQC data base for a 20-year period. The data was filtered based on ICD 10 and CHOP codes for isolated lateral malleolar fractures to compare the in-hospital outcome between HVCs and LVCs. We found significant differences in patient characteristics and short-term outcomes between the HVC group and the LVC group.

One strength of this study is the large sample size, which allowed controlling for different cofactors. Another strength is that the AQC databank provides prospective collected and standardised data. However, there are certain limitations. The de-identified patient information from the AQC only assesses in-hospital data and complications, missing relevant events after discharge. Furthermore the de-identified dataset made it impossible to re-check entered data, add missing relevant information. In addition, the data quality depends on the physician who input it and cannot be controlled. Moreover, due to anonymisation additional information about the treating hospital is not available. Also, the overestimation of biostatistical significance, easily reached due to the large sample size, is problematic and must be interpreted carefully in a clinical context.

Comparing HVCs and LVCs, the younger age of patients, the predominance of male patients and the more frequent use of external fixation in the HVC group could be seen as a consequence of the bipolar incidence of malleolar fractures, having a peak with young male patients mostly due to high-energy trauma and a second peak in elderly (mostly female) patients due to lower-energy trauma [3, 23]. This is also consistent with the higher rate of open fractures at HVCs.

Regarding the discrepancies in the in-hospital outcomes, patients at HVCs had a longer hospitalisation period and duration of surgery and a higher complication rate. The longer hospitalisation at HVCs with longer pre- and post-operative stays could be caused by a structurally induced delay at the HVCs (waiting lists for surgery) and the increased use of external fixation, which often necessitates a second procedure. The use of external fixation also indicates a potentially worse soft tissue condition and more complex fracture patterns due to the predicted high-energy trauma in the younger patient population. In particular, the associated soft tissue injury is a risk factor for longer hospitalisation and complications [24, 25]. A slightly shorter postoperative stay was noted in the top-10% centres, despite the more frequent use of external fixation. This might indicate, together with the lower rate of discharge to home, a tendency for very HVC to discharge patients to regional hospitals or geriatric clinics.

Furthermore the higher rate of privately insured patients in HVC could be a confounding factor, which was accounted for in multivariate analysis in our study population. Yet it has been found to be associated with higher complication rates in previous studies at a Swiss trauma centre [26].

Despite the difference in hospitalisation duration between HVCs and LVCs, the mean LOS ranks at the lower end of the described LOS in the literature [23, 27–31]. Regarding the differences in the LOS, a multicenter analysis from the Netherlands demonstrated longer hospitalisation at HVCs for fractures of the lower extremities. This was attributed in particular to the severity of the trauma (which is probably also the cause in our study) and the accompanying injuries [32]. Jain et al. showed a decreased LOS with higher surgeon case load for humerus fractures [33]. To the best of our knowledge, other data comparing the influence of hospital volume on LOS for other fractures is not available. For planned orthopedic procedures, such as hip and knee arthroplasty, there is a shorter LOS at HVCs and a higher case load per surgeon [34–38].

We found an overall complication rate of 2.5%. The LVCs showed a complication rate of 1.9% compared to 3.2% for HVCs (*p *= 0.001). Nonetheless, these complication rates are at the lower end of the reported complication rates for malleolar fractures in the literature, ranging from 2–14% [39–42] and even up to 26% in elderly subpopulations [43]. A higher mortality rate did not occur in this study. These discrepancies in in-hospital outcomes may be due to factors such as institutional differences, different patient characteristics, injury severity or the surgeon’s experience. It should be noted that our data cover only in-hospital follow-up and therefore misses complications during the outpatient phase. Accuracy of data collection is also a factor. Some of these variables can be accounted for in our study but not all. At HVCs we observed a younger and predominantly male patient collective, which leads to the assumption of high-energy trauma as the cause of injury and more complex fracture patterns [23], resulting in a higher complication rate.

In general, teaching is a risk factor for a worse outcome. The significantly higher frequency of teaching operations at HVCs was associated with prolonged LOS, which is consistent with observations for teaching procedures in relation to hip fractures [12, 44] and general surgeries [45]. Otherwise, a large databank study of emergency and general surgeries found no difference in LOS between teaching and nonteaching surgeries [46]*.*

Another acknowledged factor in extended hospitalisation seems to be the prevalence of comorbidities as an independent risk factor [47]. In our study, a higher prevalence of comorbidities in the HVC group was noticeable, as was a prolonged stay. Additionally, in a National Health Service (NHS) investigation of ankle fractures a prolonged stay was associated with the primary use of external fixation [29], which was five times more frequent at HVCs in our study.

The discussion regarding whether there is a difference in outcome between HVCs and LVCs has been evaluated for different surgical disciplines in the literature. Specifically for traumatology, three papers evaluated the outcomes at HVCs regarding the influence of caseload on clinical outcomes. In a single-center observation by Sava et al., the authors saw no change in mortality in relation to hip fractures based on the surgeon caseload [48]. Similarly, a meta-analysis by Wiegers et al. of hip fracture treatment at HVCs and LVCs found no significant difference in outcomes in general [49]. However, Marx et al. found caseload to be a significant predictor of in-hospital mortality [50]. Dumas et al. found evidence for other surgical disciplines and procedures. For example, they found advantageous outcomes of tracheotomies as small, routine, emergency procedures at HVCs [51]. Amato et al. found advantages for HVCs for 26 clinical areas, including hip fractures [52]. Regarding the situation in Switzerland, Mehra et al. found a preferable outcome and economic advantages in hospitals with higher structural care levels for femoral neck fractures. Correlating data for ankle fractures is currently not available.

## Conclusion

There is no evidence regarding the influence of hospital volume on in-hospital mortality for patients with ankle fractures. Despite this, there is evidence of a prolonged LOS and a higher complication rate associated with treatment at HVCs for ankle fractures. Whether this is a function of the processes and treatment strategies or the underlying patient characteristics cannot be definitively answered. Teaching procedures were shown to be safe with no association with higher mortality or complication rates. However, further research is needed, particularly due to the high number of cases and the total cost.
